# Site- and Zone-Dependent Changes in Proteoglycan Content and Biomechanical Properties of Bluntly and Sharply Grooved Equine Articular Cartilage

**DOI:** 10.1007/s10439-022-02991-4

**Published:** 2022-06-26

**Authors:** Ali Mohammadi, Nikae C. R. te Moller, Mohammadhossein Ebrahimi, Saskia Plomp, Harold Brommer, P. René van Weeren, Janne T. A. Mäkelä, Juha Töyräs, Rami K. Korhonen

**Affiliations:** 1 Department of Applied Physics, University of Eastern Finland, Kuopio, Finland; 2 Department of Clinical Sciences, Faculty of Veterinary Medicine, Utrecht University, Utrecht, The Netherlands; 3 Research Unit of Medical Imaging, Physics and Technology, Faculty of Medicine, University of Oulu, Oulu, Finland; 4 School of Information Technology and Electrical Engineering, The University of Queensland, Brisbane, Australia; 5 Science Service Center, Kuopio University Hospital, Kuopio, Finland

**Keywords:** Articular cartilage, Chondral defect model, Horse, Proteoglycan, Stress–relaxation, Osteoarthritis (OA), Digital densitometry

## Abstract

In this study, we mapped and quantified changes of proteoglycan (PG) content and biomechanical properties in articular cartilage in which either blunt or sharp grooves had been made, both close to the groove and more remote of it, and at the opposing joint surface (kissing site) in equine carpal joints. In nine adult Shetland ponies, standardized blunt and sharp grooves were surgically made in the radiocarpal and middle carpal joints of a randomly chosen front limb. The contralateral control limb was sham-operated. At 39 weeks after surgery, ponies were euthanized. In 10 regions of interest (ROIs) (six remote from the grooves and four directly around the grooves), PG content as a function of tissue-depth and distance-to-groove was estimated using digital densitometry. Biomechanical properties of the cartilage were evaluated in the six ROIs remote from the grooves. Compared to control joints, whole tissue depth PG loss was found in sites adjacent to sharp and, to a larger extent, blunt grooves. Also, superficial PG loss of the surgically untouched kissing cartilage layers was observed. Significant PG loss was observed up to 300 *µ*m (sharp) and at 500 *µ*m (blunt) from the groove into the surrounding tissue. Equilibrium modulus was lower in grooved cartilage than in controls. Grooves, in particular blunt grooves, gave rise to severe PG loss close to the grooved sites and to mild degeneration more remote from the grooves in both sharply and bluntly grooved cartilage and at the kissing sites, resulting in loss of mechanical strength over the 9-month period.

**Supplementary Information:**

The online version contains supplementary material available at 10.1007/s10439-022-02991-4.

## Introduction

Articular cartilage is a soft connective tissue covering the ends of articulating bones that accommodates and partially absorbs the forces generated by locomotion and facilitates smooth movements in diarthrodial joints. This biphasic soft tissue consists of sparsely distributed chondrocytes (cells) in an extracellular matrix (ECM) that consists mainly of water, collagen type II, and proteoglycans (PGs).^30,38^ This specific composition and organization provide its unique viscoelastic mechanical behavior that combines great compressive and shear strength with resilience.

Osteoarthritis (OA) is the most common form of joint disease in humans and horses.^4,39,43,56^ It is characterized by progressive and irreversible deterioration of articular cartilage and alterations in the joint's bone and soft tissues.^5,22^ Post-traumatic osteoarthritis (PTOA) is a common type of OA,^2^ that occurs as a sequel to damage of the articular tissues or joint instability caused by an acute injury.^27^ PTOA, unlike idiopathic OA, often occurs in young patients and often progresses quickly.^54^ However, the initiation and progression of PTOA are poorly understood.

In order to better understand the pathophysiology of PTOA, especially in its early stages, and to study the effects and mechanisms of treatment, several models of traumatic OA in animals have been used.^12,34,47^ One of these previously described models is the groove model. In this model, trauma of the hyaline, non-calcified cartilage layer is induced surgically to study the progression of degenerative changes in the joint.^7,35,36^

In our previous study,^37^ we investigated the structural, compositional, and functional effects of blunt and sharp cartilage damage on the joint. In that study, microscopical analysis (optical density evaluation) was limited to grooved cartilages and measuring the mean value of PG content around the lesions. That analysis showed significant local PG loss around both types of grooves compared to contralateral sites. On macroscopic and microscopic evaluation there were no differences observed between the areas of cartilage opposing the grooves (“kissing sites”) and the same areas in contralateral, non-grooved joints.

In order to obtain a better understanding of the effect of sharp and blunt injuries on the deterioration of articular cartilage, a more comprehensive microscopical evaluation was deemed necessary. This evaluation should include estimating PG content in regions that are remote to the grooves and, more interesting at the kissing sites. In addition, it should include analyzing PG content changes as a function of tissue depth or distance from the groove into the surrounding tissue instead of using a mean value (the mean optical density of the ROIs). This is highly relevant because it is known that during OA progression, PG loss, in combination with the disruption of the superficial collagen network, and increased interstitial fluid content,^8,26^ affects the load-bearing capacity of the tissue. It has even been suggested that the PG loss occurs before collagen matrix damage,^19,23^ and that changes in the collagen network organization are minor around cartilage lesions and more significant at the cartilage-bone interface.^51^

In the present study we aimed to gain further insights into the subtle events taking place at the early stages of cartilage degeneration in bluntly and sharply grooved joints. Also, we aimed to provide novel data that could be combined with the collected data in our previous study^37^ for computational modelling, in order to unravel the tissue deterioration mechanisms in grooved joints. Thus, we investigated long-term changes in PG distribution of bluntly and sharply grooved equine carpal cartilage and the opposing contact surfaces as a function of tissue depth and distance-to-groove. Biomechanical measurements were also used to analyze changes in the elastic properties of cartilage. We hypothesized that the shape and size of the groove could significantly affect the rate and extent of cartilage deterioration meaning blunt grooves will induce higher local PG loss around the grooves compared to sharp grooves, which may affect the remote area of cartilage. Moreover, we hypothesized that these changes do not occur in the kissing cartilage surfaces.

## Materials and Methods

### Animal Model

The Utrecht University Animal Experiments Committee and the Central Committee for Animal Experiments (permit AVD108002015307), in compliance with the Dutch Act on Animal Experimentation, approved the study.

**Figure 1:**
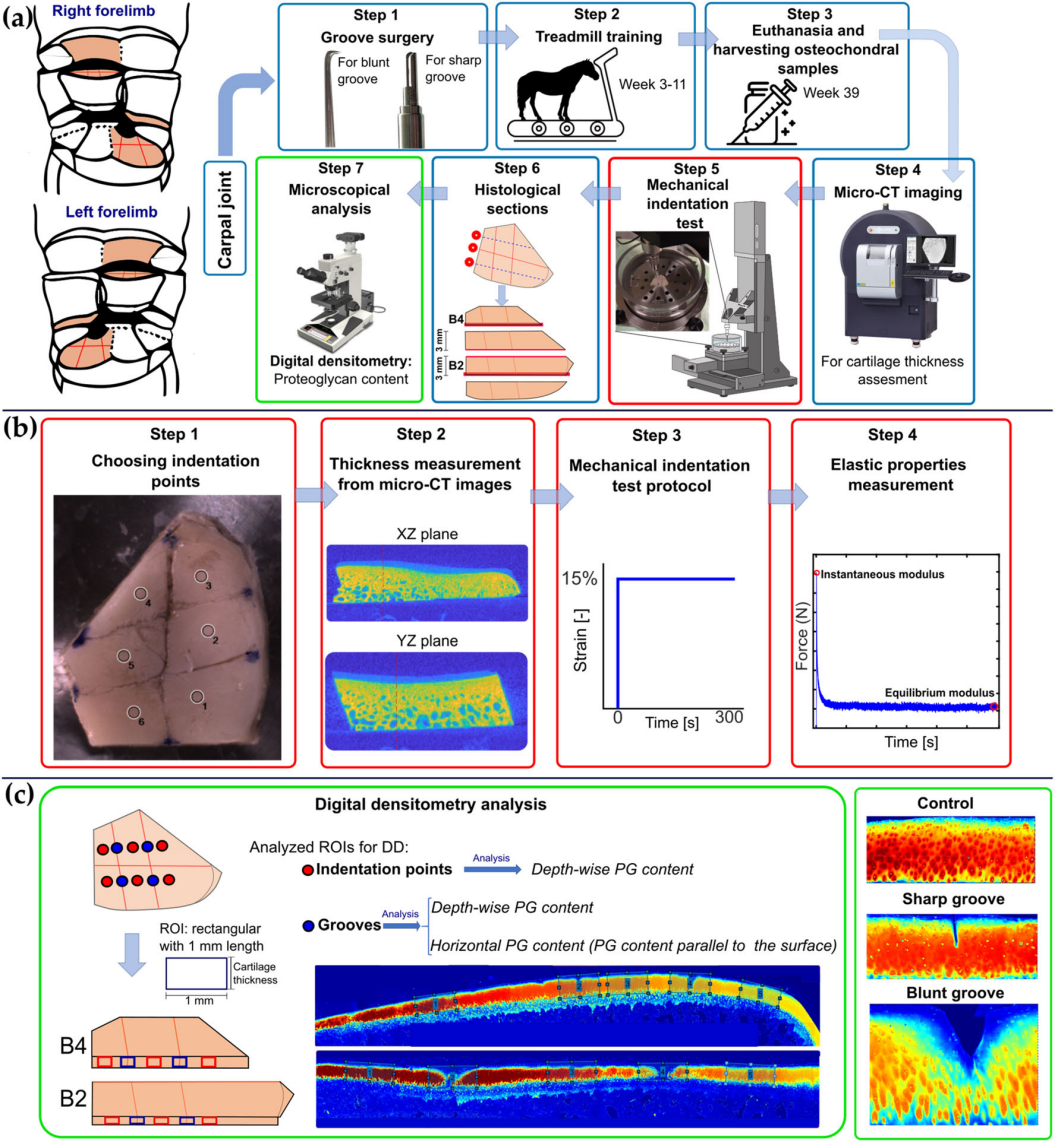
The workflow of the study. (a) An illustration of the different steps performed in this study, including surgery, duration of exercise program, harvesting samples, micro-CT imaging, mechanical testing, making histological sections (the bold red lines are histology sections), and microscopical analysis (digital densitometry), and (b) the biomechanical test steps including indentation points selection in the mechanical testing machine, finding the thickness of selected points from micro-CT images, and performing the mechanical indentation protocol, indicating points where the moduli were calculated, and (c) digital densitometry (DD) analysis steps including choosing the regions of interest (ROIs) and calculating the vertical and horizontal profiles of PG content as well as representative images of PG content in control, sharp, and blunt grooved cartilage.

Nine healthy adult female Shetland ponies (6.8 ± 2.6 years; range 4–13 years) were used for this study. The number of ponies was chosen after a power analysis (power 0.9 and *p* < 0.05) based on a pilot study^52^ and previous groove model studies.^7,34,36^ Briefly, blunt and sharp grooves were made by arthrotomy, performed by a European board‐certified equine surgeon (HB) in one randomly assigned front limb per animal. Three grooves (two in parallel in palmaro-dorsal direction and one in mediolateral direction) were randomly made in the cartilage of the radial facet of the third carpal bone (middle carpal joint) and of the intermediate carpal bone (radiocarpal joint), as described previously^37^ (Fig. 1a, step 1). Blunt and sharp grooves were randomly assigned to each of the two cartilage layers. The contralateral joints were sham-operated and used as healthy control joints.

After three weeks of box-rest, the ponies were subjected to an incremental exercise protocol for 8 weeks. After 26 weeks, they were allowed free pasture exercise. Samples were collected and prepared as described in previous publications.^37,46^ Briefly, animals were sacrificed 39 weeks after the groove surgery (euthanasia after induction of general anesthesia), and both grooved and control joints were harvested and stored at − 20 °C (Fig. 1a, steps 2 and 3). Later, the joints were thawed overnight at 4 °C, and osteochondral samples from grooved and kissing sites in both grooved and contralateral joints were harvested using an oscillating saw (multitool PMF 220CE; Bosch) and stored at − 20 °C for the following experiments (Fig. 1, step 4–7).

### Micro-CT Imaging

Samples were thawed at room temperature and imaged using high-resolution micro-CT (Quantum FX®; Perkin Elmer) in order to measure the cartilage thickness, required for mechanical indentation testing (Fig. 1a, step 4). It should be noted that in our previous study,^37^ the micro-CT images were used for bone analyses. Imaging of osteochondral samples was conducted in the air by placing one sample at a time in a sealed plastic holder containing a wet paper towel to prevent dehydration. The micro-CT images were acquired with the following parameters: X-ray tube voltage = 90 kV, X-ray tube current = 200 *μ*A, field of view = 20 × 20 mm, isotropic voxel size = 40 *μ*m, and image acquisition time = 120 s. In-built machine software was used to transform projections to 3D reconstructed files. Samples were stored at − 20 °C until the mechanical indentation test.

### Semi‐automated Mechanical Indentation Testing of Cartilage

Samples were thawed at room temperature, and six measurement locations were selected for each sample at the cartilage surface. In grooved samples, these were three points each on the dorsal and palmar side of the groove running in mediolateral direction (Fig. 1b, step 1). The locations were the same for the kissing sites and the contralateral control samples, based on an estimated virtual groove. The sample thickness was measured at each location from the cartilage surface to the calcified cartilage, perpendicular to the surface, using the micro-CT images and a custom-made MATLAB (R2018b, Mathworks, Natick MA, USA) code (Fig. 1b, step 2).

The indentation tests were conducted using a Biomomentum Mach-1 v500css (Biomomentum Inc., Laval, Quebec, Canada) equipped with a 70 N multiaxial load-cell and a non-porous spherical tip indenter (*d* = 0.5 mm, MA034, Biomomentum). The indenter was small enough to minimize effects from the groove edges.^50^ Before the measurement, the bone of the samples was flattened using sandpaper (Mirox P80, Mirka Oy, Uusikaarlepyy, Finland) and then glued (cyanoacrylate) to the bottom of a transparent acrylic chamber filled with phosphate-buffered saline (PBS).

The machine's mapping toolbox software was utilized to map the cartilage boundaries and locate the six measurement points remote from the grooves. Moreover, the Mach-1 Motion software was used to define the test parameters, including indentation amplitude, indentation rate and relaxation time. During the indentation test, the device precisely maps the surface geometry by obtaining the surface angle based on coordinates (*x*, *y*, and *z*) of 4 surrounding points, 1 mm normal distance from the test point. Then, indentation occurred in the vertical (*z*) direction, and by using the surface angle, the vertical force and displacement were converted to the component normal to the surface at the measurement point by the software.^57^

A one-step stress–relaxation test perpendicular to the surface was applied to each point with 15% of the strain of cartilage thickness (amplitude) and a strain rate of 100%/s, followed by 300 s of relaxation.^9,31,33^ The required time to reach equilibrium (relaxation rate at the end of the measurement < 0.1 Pa/min) was selected based on pilot tests.

This one-step stress–relaxation protocol was selected, first, to retain the sample in the linear region (15%) and avoid damaging it,^3^ and second, to obtain the instantaneous and equilibrium moduli in the shortest time (one step). Since we had six measurement locations in each sample, a more extended measurement protocol and submersion in room temperature PBS could have led to an increased risk of tissue damage and/or alteration of tissue properties (e.g., proteoglycan loss), respectively. Third, the fast loading rate was chosen to reach high fluid pressurization and collagen-fluid interaction in the sample during loading.

Equilibrium and instantaneous Young’s moduli were calculated from the measured equilibrium (equilibrated) and instantaneous (peak) stress/strain ratios, respectively, and corrected using the Hayes equation, which considers the measurement geometry.^16^ Poisson’s ratios were set at 0.2 and 0.5 for the equilibrium and instantaneous responses, respectively.^9,25,31^

### Microscopic Analysis

After the biomechanical measurements, osteochondral samples were cut at 6 mm from the (virtual) central groove towards the dorsal and palmar sides. They were fixed in 10% formalin (Riedel‐de Haen 33220) and decalcified for 10 weeks. Then, samples were cut into 4 parts (Fig. 1a, step 6), dehydrated in graded ethanol solutions and embedded in paraffin. Three adjacent 5 micrometer thick sections from the locations corresponding to the biomechanical indentation test points were cut with a microtome (Fig. 1a, step 6) and stained with Safranin-O/Fast-Green.

Digital densitometry (DD) imaging was acquired with a light microscope (Nikon Microphot FXA, Tokyo, Japan) equipped with a CCD cooled camera (Hamamatsu photonics K.K, Hamamatsu City, Japan, pixel size = 0.14 *μ*m) and 4x magnification. The images were calibrated against neutral density filters (optical density values 0.0, 0.3, 0.6, 1.0, 1.3, 1.6, 2.0, 2.6 and 3.0) (Schott, Mainz, Germany). Three slices per location were measured. The optical density of the images (that reflects fixed charged density of proteoglycans^24^) was used to estimate the PG distribution in the tissue. The results are presented in arbitrary units (AU).

DD analyses were conducted for 10 regions of interest (ROIs) per surface (Fig. 1c) with a width of 1 mm using a custom-made code in Matlab software (R2019, Mathworks Inc., MA, USA). These ten ROIs included the six biomechanical indentation locations (from here, we mention these points as points remote to the grooves) (Fig. 1c, red points) and 4 locations at the (virtual) grooves running in palmaro-dorsal direction (Fig. 1c, blue points). To calculate depth-wise PG content, in each ROI, pixel values were averaged in the horizontal direction across the whole tissue depth. To allow comparison between the samples, the thickness of the tissue was normalized (0 = cartilage surface and 100 = cartilage-bone interface).

The PG content as a function of distance from the groove was determined in the 4 locations at the (virtual) grooves for grooved samples and their contralateral controls. To estimate this, the middle part of the (virtual) groove was found, and the pixel values from the groove to 0.5 mm distance in both directions were averaged in the vertical direction (Fig. 1c, blue ROIs). To allow comparison between the samples, the 0.5 mm in each direction was divided into 50 points (each one 0.01 mm).

### Statistical Analysis

The dependent variables (i.e., elastic material properties as well as PG content) were compared between groups using linear mixed-effects (LME) models. Data from grooved sites and data from kissing sites were analyzed separately. For both datasets, ponies were selected as a mixed (random) effect and the type of injury (blunt, sharp, control) was set as a fixed variable.

To establish the relationships between the composition and mechanical parameters, the alterations in mechanical parameters were compared to the corresponding alterations in tissue composition of the superficial zone (average composition over 0–15% of the superficial zone, which is hypothetically relevant to 15% strain applied for obtaining the mechanical parameters). Moreover, Spearman’s correlation analysis was conducted to evaluate the potential relationships between the depth-wise PG content values (0–5, 0–10, 0–15, 0–20, 0–50, 0–100% of tissue thickness) and the elastic material parameters of cartilage. All statistical analyses were performed using SPSS Statistics version 27.0 (IBM), and *p* < 0.05 was set as the level of statistical significance.

## Results

### Depth-Wise Proteoglycan Alterations

**Figure 2:**
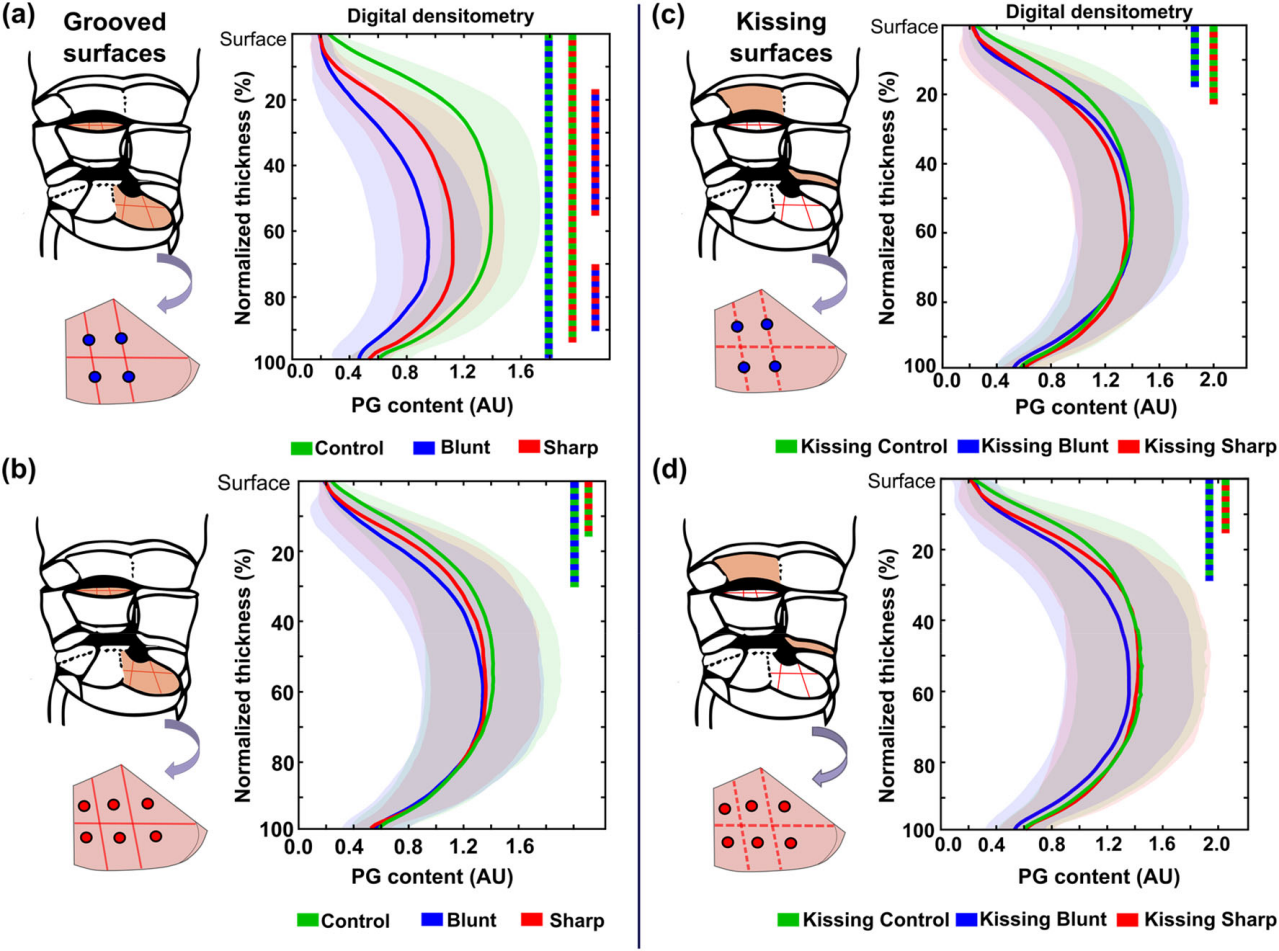
Depth-wise PG content (arbitrary unit) in areas adjacent to (blue dots) and areas in between the grooves (red dots) for (a, b) grooved and (c, d) kissing surfaces. For the grooved surfaces, the sites were grouped to the locations (a) adjacent to the grooves and (b) remote from the grooves. Similarly, the kissing surfaces were grouped to the locations (c) adjacent to the virtual grooves and (d) remote from the virtual grooves. The solid lines represent the mean of the peak values, whereas the shaded areas represent the standard deviations. Color bar at right demonstrates significant differences at each percentage of depth (*p* < 0.05) between the groups.

The cartilage adjacent to blunt grooves had a lower PG content than the cartilage adjacent to sharp grooves at 15–55% and 70–91% of the tissue depth. For both groove types, the PG content adjacent to the lesion was lower than that in control cartilage at 0–100% (blunt vs control) and 0–94% (sharp vs control) of the tissue depth (*p* < 0.05) (Fig. 2a). In addition, in ROIs remote from the blunt- and sharp grooves (indentation points), a lower PG content was observed compared to the corresponding ROIs in control cartilage at 0–30% and 0–15% of the tissue depth, respectively (Fig. 2b).

In the kissing sites of both types of grooves, the tissue adjacent to the virtual grooves had a lower PG content compared to control cartilage at 0–18% (kissing blunt vs kissing control) and 0–21% (kissing sharp vs kissing control) of the tissue depth (Fig. 2c). Moreover, we found superficial PG loss in the ROIs remote from the virtual grooves of both kissing blunt (0–27% of the tissue depth) and kissing sharp (0–15% of the tissue depth) cartilage compared to their corresponding controls (Fig. 2d).

### Proteoglycan Content as a Function of Distance from the Groove

**Figure 3:**
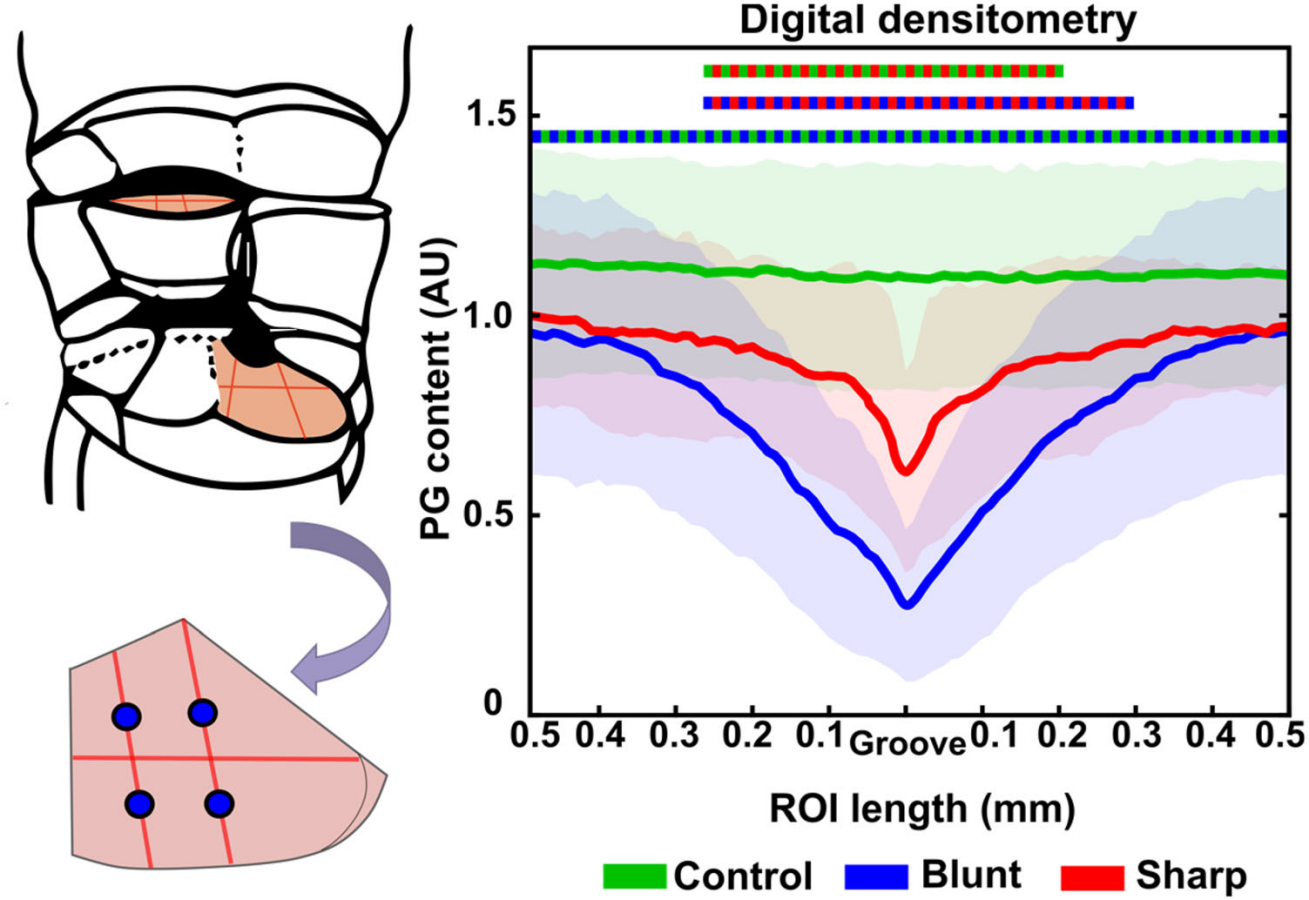
PG content (arbitrary unit) as a function of distance from the groove into the surrounding tissue in sharply and bluntly grooved cartilage. The solid lines represent the mean of the peak values, whereas the shaded areas represent standard deviations. Color bar at the top demonstrates significant differences at each point (0.01 mm) between the groups (*p* < 0.05).

The amount of PGs in the bluntly grooved cartilage gradually increased with increasing distance from the groove but remained lower than controls over the entire ROI (0.5 mm from the groove in both directions). Sharply grooved cartilage showed a similar pattern, but the difference from control cartilage became insignificant at around 0.3 mm distance from the groove (Fig. 3).

### Composition-Function Analysis with the Elastic and Viscoelastic Mechanical Parameters

**Figure 4:**
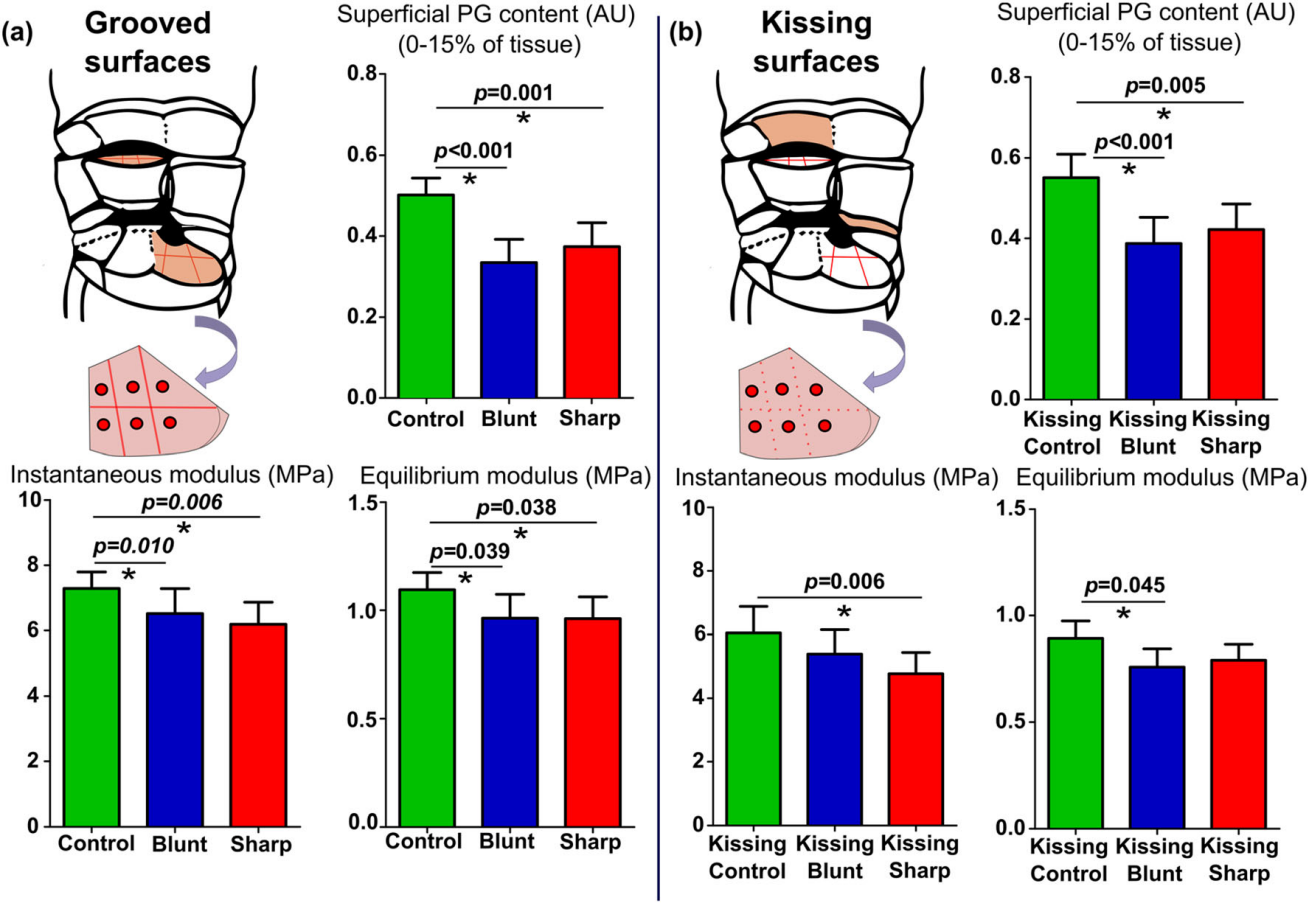
Observed changes in the superficial PG content (arbitrary unit) (0–15%) and biomechanical properties (equilibrium and instantaneous moduli in MPa) in (a) grooved cartilage and (b) kissing cartilage. Boxplots represent the mean, and the 95% confidence intervals (CI) are shown as error bars.

The cartilage at the remote locations in the blunt and sharp grooves had a lower superficial (0–15% of tissue) PG content compared to the controls (Fig. 4a). Similarly, the grooved cartilages had lower equilibrium and instantaneous moduli than in control samples (*p* < 0.05) (Fig. 4a). Compared to kissing cartilages on the contralateral joint, the kissing sites of blunt and sharp lesions (‘kissing blunt’ and ‘kissing sharp’) had a lower superficial PG content (*p* < 0.05) (Fig. 4b). Biomechanical test results showed lower equilibrium modulus in kissing blunt cartilages and lower instantaneous modulus in kissing sharp cartilages than control samples (*p* < 0.05) (Fig. 4b).

The scatter plot of biomechanical parameters and composition (PG content in different depths) is shown in Figs. S1 and S2 in the Supplementary Material. The PG content analyzed for all tissue depths showed a positive but weak linear correlation with the equilibrium modulus. For the instantaneous modulus, the corresponding correlation was only found until 20% of the tissue depth.

## Discussion

We mapped and quantified local and remote changes of PG content and biomechanical properties of grooved and kissing (not surgically damaged) articular cartilage across the joint surfaces of ponies. In the grooved cartilage, whole tissue depth PG loss was found in sites adjacent to sharp and, to a larger extent, blunt grooves. The groove-specific distance of degeneration revealed lower PG content at 300 and 500 *µ*m distance from the groove in sharply and bluntly grooved cartilage respectively. More interestingly, PG loss did not solely occur on the groove sites but also at the sites remote to the groove with more loss in case of blunt grooves. Corresponding changes were also found in biomechanical test results with lower moduli in grooved cartilage than in controls. Importantly, contrary to our second hypothesis, in both sharply and bluntly grooved joints, digital densitometry evaluation revealed the presence of cartilage deterioration in cartilage of the opposite joint surface, the so-called kissing sites.

In the quantitative gait analysis presented in our previous article,^37^ we found mildly progressive lameness in the ponies. Moreover, previous groove model studies reported gait alterations within 10 weeks after surgery.^13,34^ The gait pattern change could lead to abnormal kinetics and kinematics of motion influencing joint contact force and subsequently altering cartilage stress/strain. Earlier computational,^40^ experimental and numerical studies,^14,15^ demonstrated that changes in the biomechanical environment might be the main factor contributing to significant PG loss near the groove. Indeed, it is suggested that the excessive strain can lead to cell apoptosis and breakdown of cartilage extracellular matrix, resulting in accelerated PG loss near the grooves.^40^ Size and shape of the defect can also dramatically affect macroscopic cartilage deformation and intra-tissue strain distribution under compressive loading,^6,14^ which in this study, may have led to higher PG loss in bluntly grooved cartilages (PG loss up to 500 *µ*m distance from the groove).

The superficial PG loss at the remote and kissing sites (that were not surgically damaged) could be caused by biomechanical and/or biochemical (elevated levels of inflammatory cytokines) degradation mechanisms.^1^ Gene expression levels of inflammation markers interleukin-6 (IL-6) (week 23) and chemokine (C–C motif) ligand 2 (week 11 and 23) were elevated in synoviocytes from grooved joints compared to control joints (presented in our previous study^37^). A recent mechanobiological computational modeling study^41^ showed that the biochemical degeneration could cause superficial PG loss in the geometrically intact areas of grooved cartilage. Moreover, it should be noted that, in the tissue opposing the groove rim biomechanical factors may play a role as well, as the transition from contact to non-contact regions can cause higher local strains, and sliding may modulate mechanical wear and fatigue processes, as well as chondrocyte responses to joint loading.^14^

The equilibrium modulus correlated positively with PG content, which is in line with the strong influences of the PG on the equilibrium modulus.^42,48^ However, the weak correlation found between instantaneous modulus and PG content also indicates that other factors such as collagen content and collagen orientation may control the indentation. Therefore, in order to obtain a more comprehensive picture of the changes occurring in different locations across the grooved joint surfaces, future studies including further analyses of cartilage composition and structure (including collagen content and collagen orientation) should be carried out.

In previous groove models, de Visser *et al*.^7^ found an enhanced degeneration showing that the grooves affect the direct adjacent cartilage, already 6-weeks post-surgery in the rat knee joint groove model. They also found degenerative joint changes in the opposite (untouched) tibial and patellar joint surfaces. Moreover, in an *ex vivo* study comparing blunt and sharp trauma in bovine cartilage,^44^ a band of cell death adjacent to the blunt lesion (extending around 100 *µ*m into the tissue) was found. In an equine metacarpophalangeal joint groove model study,^34^ a single traumatic insult to the cartilage surface accompanied by exercise led to radiographic, macroscopic, and microscopic cartilage changes. In that study, however, presence of OA in non-surgically damaged areas that were reported after macroscopic evaluation, was not supported by histological data.

We chose to estimate the bulk mechanical parameters of the cartilage. These parameters do indirectly reflect the properties of tissue constituents. A relatively high strain rate compression was chosen to highlight the effect of fluid pressurization and tension of the collagen fibers during the rapid, instantaneous loading. According to the literature, the instantaneous modulus reflects the collagen network properties and is associated with the fiber network modulus and fluid pressurization in a biphasic model.^10,20,29^ We also reported the equilibrium modulus, which is an indicator of the properties of the non-fibrillar PG matrix. The relationship between the equilibrium modulus and the non-fibrillar matrix modulus has been shown extensively in the literature.^10,11,21,32^ However, a direct assessment of the non-fibrillar and fibrillar matrix properties would require computational modeling and optimization.^49^

Our study has limitations. First, we did not have a separate healthy control group. Instead, we used the sham-operated contralateral joint as the control group. In this way, we could reduce the number of experimental animals and the degree of biological variation between them and consider the effect of invasive procedures on the results. However, these also brought some limitations to our study. The sham‐operated contralateral joints may not have been entirely free from damage and structural alterations of cartilage due to the possible alterations in motion and low-grade inflammatory responses.^17,28,37,53^ In particular, the arthrotomy and repeated sampling of synovial fluid and synovium during the nine months follow-up may have induced bilateral inflammatory responses.^17,28,45,55^ Moreover, although the literature lacks information, based on clinical veterinary experience, cartilage degeneration in the carpal joint is not typically a naturally occurring process in Shetland ponies. That may be due to limited motion (likely less shear forces) of the carpal joint compared to other joints, such as the fetlock joint. Therefore, within nine months, we would not expect any notable natural cartilage degeneration in a separate healthy control group**.**

Second, evaluation at one time-point (week 39) does not provide information on how changes in PG content progress over time. That was also not the scope of the current study. However, longitudinal changes in motion capture, synovial membrane gene expression, near-infrared spectroscopy (NIRS) and radiography gathered at several timepoints following surgery of the same animals have been addressed extensively in our previous publications.^37,46^

Third, we selected 1 mm ROI for DD analysis because wider ROIs could make them overlap. On the other hand, if ROIs had been wider, we could have found the point where PG content around blunt grooves reached the normal level.

Fourth, while indentation was not necessarily conducted perfectly perpendicular to the cartilage surface, stresses and strains normal to the surface were calculated and used in the analyses.^57^ This caused minor variations in the final strains between the samples and measured locations. Yet, we were in the linear region of the equilibrium stress-strain curves, thus, this uncertainty should have negligible effect on the results.

In conclusion, the presented equine carpal joint groove study adds novel data to the previous animal models of OA with features mimicking (early) human OA. This study particularly provides detailed information of site- and location-specific variations in PG content and biomechanical properties around different types of cartilage grooves, which is essential for a better understanding of the development of PTOA. The combination of data from this study with data from the previous studies^18,37,46^ will provide valuable input for the validation of mechanical and mechanobiological computational models. These models can ultimately be utilized to elucidate the mechanisms behind the joint deterioration as a sequel to cartilage damage.

## Supplementary Information

Below is the link to the electronic supplementary material.

Supplementary file1 (MP4 49746 KB)
